# Update on Atypicalities of Central Nervous System in Autism Spectrum Disorder

**DOI:** 10.3390/brainsci10050309

**Published:** 2020-05-20

**Authors:** Ahmad Naqib Shuid, Putri Ayu Jayusman, Nazrun Shuid, Juriza Ismail, Norazlin Kamal Nor, Isa Naina Mohamed

**Affiliations:** 1 Advanced Medical and Dental Institute, Universiti Sains Malaysia, Bertam, Kepala Batas 13200, Pulau Pinang, Malaysia; nqb_ahmad@yahoo.com; 2 Department of Pharmacology, Faculty of Medicine, Universiti Kebangsaan Malaysia, Cheras, Kuala Lumpur 56000, Malaysia; putri.ayujay@gmail.com; 3 Faculty of Medicine, Universiti Teknologi MARA, Sungai Buloh 47000, Selangor, Malaysia; 4 Autism Research Group, Department of Pediatrics, Faculty of Medicine, Universiti Kebangsaan Malaysia, Cheras, Kuala Lumpur 56000, Malaysia; juriza@ppukm.ukm.edu.my (J.I.); norazlyn@ppukm.ukm.edu.my (N.K.N.)

**Keywords:** autism, autism spectrum disorder, central nervous system, brain

## Abstract

Autism spectrum disorder (ASD) is a heterogeneous, behaviorally defined, neurodevelopmental disorder that has been modeled as a brain-based disease. The behavioral and cognitive features of ASD are associated with pervasive atypicalities in the central nervous system (CNS). To date, the exact mechanisms underlying the pathophysiology of ASD still remain unknown and there is currently no cure or effective treatment for this disorder. Many publications implicated the association of ASD with inflammation, immune dysregulation, neurotransmission dysfunction, mitochondrial impairment and cell signaling dysregulation. This review attempts to highlight evidence of the major pathophysiology of ASD including abnormalities in the brain structure and function, neuroglial activation and neuroinflammation, glutamatergic neurotransmission, mitochondrial dysfunction and mechanistic target of rapamycin (mTOR) signaling pathway dysregulation. Molecular and cellular factors that contributed to the pathogenesis of ASD and how they may affect the development and function of CNS are compiled in this review. However, findings of published studies have been complicated by the fact that autism is a very heterogeneous disorder; hence, we addressed the limitations that led to discrepancies in the reported findings. This review emphasizes the need for future studies to control study variables such as sample size, gender, age range and intelligence quotient (IQ), all of which that could affect the study measurements. Neuroinflammation or immune dysregulation, microglial activation, genetically linked neurotransmission, mitochondrial dysfunctions and mTOR signaling pathway could be the primary targets for treating and preventing ASD. Further research is required to better understand the molecular causes and how they may contribute to the pathophysiology of ASD.

## 1. Introduction

Autism was first described by a psychiatrist named Leo Kanner based on his observations on 11 children with severe communication problems, repetitive behavior, and acute lack of social interaction. Kanner’s original description has led to the recognition of autism as a disorder decades later [1]. It is part of a broader range of conditions known as autism spectrum disorder (ASD). The term autism and ASD are used interchangeably. ASD is defined as a complex neurodevelopmental disorder characterized by impairments in social interactions and communication, as well as by the presence of purposeless repetitive behaviors and restrictive interests [2]. Individuals with ASD have difficulty in expressing and understanding certain emotions and moods, abnormal eye contact, restricted ways of using toys, preferences of isolated play and minimal changes to routine. These characteristics have made it difficult for them to establish relationships with others, act in an appropriate way and live independently [3].

Developmental disabilities ranging from mild disabilities such as speech and language impairments, to more serious developmental disabilities such as intellectual disabilities, cerebral palsy and autism, have been identified in approximately 1 in 6 children in the United States. The prevalence of ASD has dramatically increased in the last few decades at the rate of 1 in 2500 children around 1980s to 1 in 150 children in 2007 [4]. According to the data from the Autism and Developmental Disabilities Monitoring (ADDM) Network of the US Centers for Disease Control and Prevention (CDC), approximately, 1 in 68 children have been identified with ASD from 2010 to 2012 [5]. For 2014, the overall prevalence of ASD has increased to 16.8 per 1000 (1 in 59) children aged 8 years [6]. The rate of ASD diagnosis was four times more common in males than females [7]. The increase in ASD prevalence was partly attributed to the increased awareness and reporting practice of the disorder, as well as an improved diagnostic criteria [8].

Approximately, 90 percent of ASD cases have been classified as idiopathic, while about 10 to 20 percent were caused by known genetic etiology [9]. In recent years, intense scientific works have revealed that ASD is genetically driven with heritability indices estimated at 85 to 92 percent and could be triggered by environmental risk factors especially those influencing fetal and early-life development [10,11]. The symptoms of autism appeared before 36 months of age, while regression or loss of skills usually occurred between 18 and 24 months in 30 percent of the affected children [12]. It may persist throughout life, often in a more muted form [2]. In fact, ASD has affected more children than diabetes, acquired immune deficiency syndrome (AIDS), cancer, cerebral palsy, cystic fibrosis, muscular dystrophy and Down’s syndrome combined [13]. The Global Burden of Disease Study 2010 reported that the global prevalence and burden of disease for ASD in 2010 was 1 in 132 individuals, which translated to 52 million cases of ASD and 7.7 million disability-adjusted life-years (DALY) across the globe [14]. Among the mental disorders, ASD was the leading cause of disability in children under 5 years of age in terms of years lived with disability (YLDs). ASD was also ranked among the 20 leading causes of disability for the under 5-year age group. These data indicated that ASD is accounted for indisputable health loss across the lifespan.

The proposed pathogenesis of ASD comprises many distinct mechanisms including chronic neuroinflammation, gamma-aminobutyric acid (GABA) imbalance, monoaminergic dysregulation and mitochondrial dysregulation [15]. However, the precise mechanism underlying the pathophysiology of ASD remained unknown and currently, there is no cure or effective treatment for this disorder. Major challenges toward finding an effective cure for this disorder include heterogeneity of its etiology and the lack of consistent and reliable genetic or biologic diagnostic markers for accurate classification and early diagnosis of ASD [16].

There have been many hypotheses for ASD etiology. For instance, there are emerging evidence that neurotransmitter imbalances, gut-brain axis abnormalities and maternal infection may be involved in the pathogenesis of ASD. Nevertheless, ASD appears to be resulted from developmental factors that centrally affect functional brain system. Understanding the pathophysiology of ASD also requires analyzing brain structures and processes associated with these disorders. Hence, this review paper anticipated the association between changes in the CNS and ASD. Despite the substantial number of observations made concerning the cellular and molecular dysfunctions associated with ASD, the basic central mechanism of these disorders that summarizes the major physiological abnormalities of CNS has not been reviewed. Brain development aberration, neuroimmune alterations, neurotransmission dysregulation, mitochondrial dysfunction and cell signaling pathway dysregulation are among the major etiological components in ASD. This review discusses the accumulating literatures on ASD, giving special attention to the brain structure and function abnormalities, neuroglial activation and neuroinflammation, glutamatergic neurotransmission dysfunction, mitochondrial dysfunction and mechanistic target of rapamycin (mTOR) signaling pathway dysregulation in the development of ASD. In addition, it also focuses on the molecular and cellular factors for the pathogenesis of ASD and summarizes recent studies that examined how they might have affected the development and function of the CNS.

## 2. CNS and Social Function

Vast parts of brain regions, which are made of the neural circuitry, are involved in various aspects of social cognition and perception. Social cognition is referred to as the fundamental ability to perceive, categorize, remember, analyze, reason with and behave towards others [17]. The ability to perceive is not only dependent on vision and hearing, but also sensation (sense of smell and somato-sensation). It also depends on the connection with memories and emotions in the amygdala-hippocampal complex and other limbic structures [18]. Meanwhile, social response formation involves automatic, stereotyped motor patterns encoded in brain stem nuclei, hypothalamus, central limbic and medial temporal structures, which interplay with the frontal cortex. Some parts of the cerebellum and the corpus callosum are also important for the “social brain”. The monoaminergic neurotransmitter systems that are involved in the functioning of the “social” circuits and controlling the activity in vast areas of the brain comprise the serotogenic, mesolimbic dopaminergic and norepinephrinergic systems. In addition, the GABAergic anti-excitatory system, peptidergic systems and neurons under the influence of steroid hormone are all essential for social functioning [18].

ASD has been linked to abnormal social brain function and neurological disorder [19]. As a disorder that features profound deficits in several aspects of social perception and cognition, neuroanatomical structure of the brain has become the focus in understanding brain mechanisms in research related to ASD. Additionally, ASD is characterized through behavioral and cognitive features that are predominantly thought to be as a result of atypical development of the brain itself.

## 3. Central Nervous Changes and ASD

Autism is also referred to as an early-onset disorder of the developing CNS [20]. Although the underlying mechanisms remain largely unknown, autism is commonly described as a brain-based disorder since many documented changes are registered in the brain [21]. In fact, the symptoms of ASD have been associated with pervasive atypicalities in the CNS [22].

### 3.1. Brain Structure and Function Abnormalities in ASD

Certain brain regions including the limbic system, particularly the hippocampus, amygdala and cerebellum, have been implicated in the pathophysiologic mechanisms and clinical expressions of the disorder [23]. Evidence from neuroimaging and postmortem studies has revealed structural abnormalities in those regions of the brain. Hypothetically, the core abnormalities in the pathogenesis of autism are located in the amygdala, adjacent limbic structures and corpus callosum [18].

The amygdala is a collection of nuclei that lies beneath the uncus of the temporal lobe at the anterior end of the hippocampal formation and the inferior horn of the lateral ventricle of the brain [24]. It influences drive-related behavior and related emotions. Amygdala stimulation is commonly followed by fear emotion, while bilateral destruction of amygdala causes reduced aggression. Amygdala deficit in autism might lead to abnormal fear responses in children; they may either show too little or too much fear compared to non-autistic children [24]. The hippocampus is also the key component of the neural system and one of the most thoroughly studied areas of the mammalian CNS. It mediates the emotion perception and regulation, and hence is also thought to be involved in the pathophysiology of autism [25].

Studies have shown that the damage to the amygdala is associated with impairments in social cognition and interpretation of emotions [26]. Abnormal patterns of the amygdala and hippocampal development were found during childhood and adolescence phases of autistic cases. In a previous study by Pierce, et al. [27], structural and neurofunctional activities in the brain regions related to face processing were evaluated using functional magnetic resonance imaging (MRI). The study revealed a significant decrease in amygdala volume in autistic adults compared to normal control subjects. The study is consistent with an earlier MRI study by Aylward, et al. [28], which demonstrated a significantly smaller amygdala volume in non-mentally retarded autistic male adolescents and young adults compared to healthy community volunteers. A significant reduction in hippocampal volume in relation to total brain volume was also noted in autistic subjects. The authors concluded that these volume reductions were related to reduction in dendritic tree and neuropil development, which likely reflected the underdevelopment of neural connections of limbic structures with other parts of the brain.

Findings of another study, however, documented that amygdala lesions did not lead to autistic symptoms [29]. The subjects were two women with developmental-onset bilateral amygdala lesions. By using comprehensive interviews, behavioral observations and widely used ASD screening questionnaires, it was found that both subjects did not exhibit autistic symptomatology despite having the amygdala lesions. This suggests that it is the abnormal connectivity between the amygdala and other structures rather than overt amygdala pathology, which contributes to ASD.

On the contrary, several studies have found that amygdala and hippocampal volumes of ASD subjects were increased from childhood to young adulthood. Three-dimensional coronal MRI measurement acquired from autistic children revealed enlargement of amygdala and hippocampi [23]. Schumann, et al. [30] showed that autistic children had larger right and left amygdala volumes than control children; however, similar changes were not seen in the adolescent group. The hippocampal volume was enlarged in all study groups. The authors speculated that amygdala is initially larger in children with autism, but they did not undergo the age-related increase in volume that normally occurs in developing children. In another study, Groen, et al. [25] measured amygdala and hippocampal volumes in a group of adolescent with autism and found significant enlargement of these parts of the brain compared to control group [25].

In a more recent study, volumetric MRI of amygdala and hippocampal subfields were measured in infant subjects with risk of ASD [31]. The authors showed significant enlargements of amygdala and hippocampi in each hemisphere and whole brain in ASD group compared to normal control. Amygdala enlargement at an early age has been related to severity of social, communication and emotional problems in ASD group [25,30]. The volumetric enlargement of amygdala and hippocampus were postulated to be an adaptive response to increased neuron activity throughout childhood and adolescence in autism. It is also plausible that the hippocampus is enlarged in response to heightened amygdala activity since the hippocampus has a regulatory role on amygdala activity through a dense network of reciprocal connections [25].

Considering this, abnormalities in those brain regions seemed to follow a different time course and the findings in adolescence and adult were quite sparse, which are summarized in Table 1. There are certainly limitations that may contribute to this discrepancy. For instance, the small sample size in some studies may result in insufficient statistical power. Several studies have included a broader age range, which may hinder the detection of developmental changes in the brain. These results need to be replicated before a definitive conclusion can be made. Nonetheless, the changes in amygdala and hippocampal structure and function reported in previous studies were in accordance with the theory that autism is caused by abnormalities of certain brain regions.

### 3.2. Neuroglial Activation and Neuroinflammation in ASD

Neuroglial cells such as astrocytes and microglia play important roles in neuronal function and homeostasis [32]. Astrocytes are star-shaped glial cells in the brain and spinal cord. Collectively, they are known as astroglia. Microglia are resident macrophage cells that are also located throughout the brain and spinal cord. Neuroglial cells are the essential regulators of immune responses in the CNS. They are also crucial in cortical organization, neuroaxonal guidance and synaptic plasticity [33]. During normal homeostatic condition, astrocytes facilitate neuronal survival by producing growth factors and mediating uptake or removal of neurotransmitters including glutamate from the synaptic microenvironment [34]. However, during astroglial activation secondary to injury or in response to neuronal dysfunction, astrocytes may secrete pro-inflammatory cytokines, chemokines and metallo-proteinases that could magnify immune reactions within the CNS [35]. Likewise, microglial activation might produce similar neuroglial responses to injury or dysfunction.

Recent evidence documented that localized inflammation in the CNS could contribute to the pathogenesis of ASD. Several studies have shown that inflammatory cytokines including tumor necrosis factor-α (TNF-α), interferon-γ (IFN-γ), interleukin-1β (IL-1β) and interleukin-12 (IL-12) were elevated in the blood mononuclear cells, plasma and serum of autistic children [36,37]. However, these findings were observed peripherally and not directly related to immune-mediated pathology within the CNS.

Vargas, et al. [12] measured 79 proteins including cytokines, chemokines and growth factors in the post-mortem brain of autistic patients to detect the presence of neuroinflammation. The levels of pro-inflammatory cytokines including transforming growth factor (TGF-β1), macrophage chemoattractant protein (MCP)-1, interleukin-6 (IL-6) and interleukin-10 (IL-10) were found elevated in brain tissues of autistic patients compared to control patients. Meanwhile, immunocytochemical analyses showed marked activation of microglia and astroglia, indicating that increased neuroglial response was part of the neuroinflammatory reactions. The authors reported that these responses could be linked to the CNS innate immune system in which microglial activation was the main cellular response to CNS dysfunction [12].

In another study, Li, et al. [38] further investigated both innate and adaptive immune responses in the brain tissue of ASD patients using Multiplex Bead Immunoassays. The results showed that proinflammatory cytokines (TNF-α, IL-6, and GM-CSF), Th1 cytokine (IFN-γ) and chemokine (IL-8) were significantly increased in the brain (cerebral cortex) extracts of ASD patients compared to the control. The high inflammatory cytokine levels were indicative of heightened immune response and may be associated with localized brain inflammation and tissue necrosis.

Taken together, peripheral immune dysregulation observed in early studies could be associated with neurotoxic events in brain. In normal condition, microglia exist in resting state and constitutively express growth factors, not cytokines or excitatory amino acids [39]. In ASD, the upregulation of peripheral chemokines/cytokines such as TNF-α, IL-6 and IL-1β might trigger the activation of microglia. Primary microglial activation can be caused by disturbances in microglial function or neuronal-microglial interactions during brain development. Meanwhile, secondary activation might be contributed by unknown factors that disturb pre- or postnatal brain development [40]. Excessive or prolonged microglial activation involving microglia-induced inflammatory cytokines and excitotoxicity might disrupt neurogenesis and neurodevelopment [41]. Table 2 describes previous studies examining neuroglial activation and neuroinflammation in ASD.

### 3.3. Glutamatergic Neurotransmission Dysfunction in ASD

Neurotransmitters generally play a major role in forming CNS and peripheral nervous system. Glutamate (Glu) is a major excitatory neurotransmitter crucial for brain development. It is important for neuronal plasticity and maintenance of cognitive function [42]. Various subtypes of glutamatergic receptor can be found on astrocytes, microglia, oligodendrocytes, neuron presynapse and axons. Glu receptor system comprises ionotropic and metabotropic glutamate receptors. Activation of these receptors is required for fast synaptic transmission, synaptic plasticity, learning and memory, motor coordination, pain transmission and neurodegeneration [39].

Major neurotransmitters including Glu, serotonin, dopamine and GABA are implicated in autism [43]. Glu has been shown to be directly involved in general cognitive functions [44]. There are compelling evidence that excess Glu could be a potent neurotoxin that leads to neuronal cell death and plays a role in the pathophysiology of some neurodegenerative diseases [45,46]. Scientific evidence has also revealed that most heterogenous symptoms of ASD are closely connected with the dysregulation of glutamatergic neurotransmission in the brain [39].

Glu levels in the brain can be measured in vivo safely and non-invasively using a neuroimaging technique called proton magnetic resonance spectroscopy (^1^H-MRS). The combination of Glu and glutamine is referred to as Glx, which represents the overall glutamate/glutamine levels and their functioning in the brain. Glu is associated with the production of oxidative energy and function of excitatory neurotransmitter, while glutamine is involved with Glu recycling and regulation of brain ammonia metabolism [47,48,49].

A study by Page et al. [50] revealed that adults with ASD had a higher concentration of Glx level in the right hippocampus compared to healthy subjects. Brown et al. [51] showed an increased Glx concentration in the auditory cortex in adults with ASD compared to healthy adult control. In other studies, high Glx concentration was also reported in the anterior cingulate gyrus of children and adolescents with ASD [47,52]. Significantly higher brain Glu levels were also detected in the brain regions of autistic children including the anterior cingulate gyrus, left striatum, left cerebellar hemisphere and left frontal lobe [53]. These brain areas were reported to be affected in ASD, causing disturbance in brain processes including cognitive, affective, sensory functions, motor task, join attention and social orienting.

In addition to brain Glu, the levels of peripheral Glu and other excitatory amino acids were also found to be elevated in autistic subjects. Hassan et al. [53] showed that blood Glu level was significantly higher in children with ASD compared to controls. Several other studies have also reported significantly higher Glu levels in plasma/platelet-poor plasma [46,54] and serum [55].

These findings are consistent with those by Aldred et al. [56], which reported significant elevations of plasma Glu in children with autism as well as in their parents and siblings. The levels of other amino acids related to glutamatergic neurotransmission such as phenylalanine, alanine, lysine, tyrosine and asparagine were significantly higher, but plasma glutamine was significantly lower in samples taken from children with autism and their parents compared to age-matched control.

In human, plasma Glu level has been seen correlated with cerebrospinal fluid Glu level, although Glu does not readily cross the blood brain barrier [45,46]. The peripheral Glu level was postulated to reflect the Glu level in the brain *per se*. The increase in Glu concentration was thought to be due to these mechanisms (i) dysfunction in enzymes that are responsible for converting glutamate to GABA [57]; (ii) lack of Purkinje cells in autistic cerebellum [58]; (iii) production of transporters that translocate Glu from endothelial cells to extracellular fluids [59] and (iv) dysfunction of the glutamate-glutamine cycle.

As for Glx level, several ^1^H-MRS studies on the brain of autistic subject have shown low Glx levels. As reported by Horder, et al. [60], adults with ASD had a significantly reduced Glx concentration in the basal ganglia, which correlated with the impairment in social communication. There were reports of significantly lower Glx concentration in the anterior cingulate cortex of adults with ASD [61,62]. In addition, two previous studies found reduced cerebral Glx [63] and Glx/creatinine ratio in the frontal lobe of children with ASD compared to healthy control [64]. These findings suggested dysfunction of the brain glutamatergic system and abnormalities of neurotransmission.

The abnormalities in Glu/Glx concentration vary depending on age group, sample characteristics and region of brain, which are summarized in Table 3. The wide age range of participants, small sample size, difference in Intelligence Quotient (IQ) level and gender difference may affect study findings. The use of psychoactive medications may also affect the findings since neurotransmitter systems are sensitive to these drugs. Recent meta-analyses of Market Research Society (MRS) data reported that metabolic abnormalities tend to decrease and normalize with age and hence are age dependent. Besides, the long duration of the illness in autistic adults may induce secondary changes to Glu levels in response to compensatory process rather than pathophysiological mechanism [46]. Since autism is a heterogeneous disorder, to avoid inconsistencies and obtain comparable and easily interpretable findings, future studies should consider using homogenous sample (age group, gender and IQ level) and avoid confounding factors including the use of medications.

### 3.4. Mitochondrial Dysfunction in ASD

Mitochondria are membrane-bound cell organelles that generate adenosine triphosphate (ATP), the energy carrier in most mammalian cells, through oxidative phosphorylation [65]. This process involves the mitochondrial electron transport chain (ETC) located in the inner mitochondrial membrane [66]. The number of mitochondria in each cell depends on the energy demands it requires. Cells with high-energy demand, including brain cells, have many mitochondria. Dendritic and axonal terminals which have important roles in ATP production, calcium homeostasis and synaptic plasticity are concentrated with mitochondria [67,68]. Cells that consume high energy are therefore very dependent on mitochondrial function [69]. Thus, abnormalities in mitochondrial function would be expected to cause brain dysfunction.

The deleterious consequences of mitochondrial disorders on neurodevelopment have attracted interest from researchers to find the possible link between mitochondrial abnormalities and autism. An immature brain is more vulnerable to defects in bioenergetics capacity since CNS is accounted for 20 percent of the body’s metabolic demand while developing neurons are dependent on oxidative phosphorylation for critical developmental processes [70]. 

Mitochondrial disorders can result in CNS dysfunction that leads to developmental regression, learning disability and behavioral disturbances [71]. The impairment of mitochondrial energy metabolism has been proposed to play a role in the pathogenesis of autism and might influence the social and cognitive deficits in ASD.

Evidence of metabolic abnormalities that indicates dysfunction of mitochondria has been described in subjects with ASD including the elevation of lactate, pyruvate or alanine level in blood, cerebrospinal fluid or brain, reduction of plasma carnitine level, abnormal level of urine organic acids and impairment of mitochondrial fatty acid β–oxidation [72]. Table 4 summarizes the findings from previous studies discussed herein. Recent studies have also reported abnormalities of lactate to pyruvate ratio [73], creatine kinase [74,75], aspartate aminotransferase (AST) [75,76], alanine aminotransferase (ALT) [76] and ubiquinone [74,77]. Several studies in subjects with ASD also discovered abnormalities in amino acids related to mitochondrial metabolism such as arginine [78], cysteine, tyrosine, serine, carnosine and β-alanine [79].

Pastural et al. [80] reported abnormalities in the biomarkers of fatty acid metabolism. Elevated levels of fatty acid elongation and desaturation of poly-unsaturated long chain fatty acids (PUFA) and/or saturated very long chain fatty acids (VLCFA)-containing ethanolamine phospholipid were observed as useful metabolic biomarkers of mitochondrial stress and autism. Elevation of saturated fatty acids and reduction of PUFA has been documented in a study by El-Ansary et al. [81], suggesting the mechanistic role of fatty acid profiles in mitochondrial dysfunction in autistic patients. Although the underlying cause of mitochondrial stress is unknown, Pastural et al. [80] postulated that glutamate, a known neurotoxin and mitochondrial disrupter, is involved in the pathophysiology of impaired mitochondrial function. The elevation of VLCFA-containing lipids levels in the plasma is indicative of mitochondrial fatty acid β–oxidation and could be causal to the pervasive CNS microglial activation observed in autism [80].

Genetic abnormalities including chromosomal abnormalities, mitochondrial DNA mutation and large-scale deletions and mutation of mitochondrial nuclear genes have been associated with mitochondrial dysfunction in ASD [82]. In an observational study by Giulivi et al. [83], children with autism were more likely to have mitochondrial dysfunction, mtDNA over-replication and mtDNA deletions than normal children. Mitochondrial dysfunction can be classified as primary or secondary in nature. The former classification occurs as a direct consequence of gene mutations, while the latter by impaired oxidative phosphorylation due to other genetic or metabolic derangement. The type of mitochondrial dysfunction responsible for autism is unknown, but it could greatly amplify and propagate brain dysfunction in autism. This was evidenced by the high incidence of mtDNA abnormalities recorded in post-mitotic tissues with high-energy demands, specifically the brain of ASD subjects [83]. Increased mtDNA copy number, mtDNA damage and deletions in leukocytes of children with ASD have also been reported in other studies [84]. A more recent study found a co-occurrence of mtDNA deletions with ASD-associated genetic alterations [85], which supports the association between mitochondrial alterations and ASD.

Direct evidence of mitochondrial dysfunction has been reported by postmortem brain tissue examinations on ASD subjects. A study by Gu et al. [86] on the frontal cortex tissues of autistic children showed reduced activities of complexes I and V with reduction in pyruvate dehydrogenase level. Other studies reported the reduction of ETC complexes (I–V) levels in the frontal [87], temporal [87,88] and cerebellar [87] areas of brain samples from autistic subjects. Taken together, it was deduced that there were impairments in the mitochondrial function and intracellular redox status in the brain of ASD subjects. The abnormalities observed in the mtDNA or ETC function may be responsible for the core features of autism.

### 3.5. Dysregulation of mTOR Signaling Pathway in ASD

Dysregulation of mTOR signaling pathway has been shown to have a pathogenic role in a variety of neurological disorders including autism. mTOR is a conserved serine-threonine kinase belonging to phosphoinositide-3-kinase (PI3K) that is crucial in the establishment of neuronal shape and size, dendritic arborization, spine morphology, axon outgrowth and synaptic plasticity in the brain [89]. mTOR is a member of two protein complexes namely mTORC1 and mTORC2 [90]. In cells, mTORC1 promotes cell growth by positively regulating anabolic processes and negatively regulating catabolic processes, whereas mTORC2 regulates cell survival, metabolism and structure by modulating downstream protein kinases and cytoskeletal elements. Altered mTOR signaling including mTORC1 hyperactivity leads to phosphorylation of numerous downstream proteins that regulate various cellular processes [91]. Therefore, perturbation of mTOR signaling cascade appears as a common pathophysiological feature of human neurological disorders.

Previous evidence on dysregulation of mTOR signaling pathway are summarized in Table 5. In a study by Tang et al. [92], postmortem analysis of superior middle temporal lobe from children and adolescents with ASD revealed increased density of dendritic spines on layer V pyramidal cells as well as aberrant mTOR activation and impaired autophagy. This brain region is particularly implicated in ASD due to its role in brain networks involved in social and communicative processes (e.g., language, social and speech perception, auditory and visual processing [93,94]. High phosphorylated-mTOR (p-mTOR) level found in the study was thought to be associated to low level autophagy in ASD patients. The findings suggested that ASD behavior and synaptic deficits during childhood and adolescent are elicited by altered mTOR signaling. However, another postmortem study has reported that the expression of mTOR and p-mTOR was decreased in fusiform gyrus samples from individual with ASD [95]. Additionally, protein levels of brain derived neurotrophic factor receptor (TrkB) isoforms and the postsynaptic organizing molecule (PSD-95) were also reduced in autistic fusiform gyrus. The study suggested that the down-regulation of Akt/mTOR pathway in autism is likely to affect spine and cortical circuits implicated in higher cognitive function and behavior, thus causing autistic phenotypes.

One recent study has examined the components of Akt/mTOR pathway in peripheral blood mononuclear cells isolated from the children with ASD. The authors showed an increased Akt/mTOR activity and higher phosphorylation of Akt/mTOR pathway proteins such as p70S6 kinase (p70S6K) and extracellular receptor kinase (ERK) in T cells of ASD children. In neurons, the Akt/mTOR pathway is believed to be important in the process of learning and memory formation by augmenting long-term potentiation of synapse [96]. Disruption in any cellular processes involved in the pathway in central nervous and immune system might impact neurodevelopment and has been connected to ASD symptoms [97,98]. Tylee et al. [99] combined gene expression studies from raw microarray data and clinical meta-data comparing individuals with and without ASD. The authors reported that genes associated with the PI3K-Akt-mTOR pathway were significantly down-regulated, whereas genes associated with RNA translation were up-regulated. In another study, Tylee, et al. [100] investigated RNA sequence of transformed lymphblastoid cells from individuals with ASD and their unaffected siblings and discovered that the genes involved in mTOR signaling were up-regulated in males subjects with ASD.

Taken together, mTOR pathway deviations in either direction were postulated to adversely affect the establishment, maintenance and function of neuronal networks that could ultimately cause autism cognitive and behavioral deficits [101]. Dysregulation of mTOR signaling pathway identified in previous studies suggests the potential mechanism in the development of ASD. Modulation of this pathway could offer benefits for syndromic autism linked to aberrant mTOR signaling.

## 4. Limitation and Challenges

As reported above, the findings of several studies were somewhat inconsistent. The discrepancies in the reported findings were expected to be due to the variable differences (age range, gender, sample size, medication and IQ level) among the performed studies. If inaccurate conclusions regarding diagnosis were made, the potential consequences would vary depending on specific factors. Some of the possible consequences include misinterpretation of results, which in turn leads to false positive or negative diagnosis. Inaccurate labeling or identification of disease that leads to false positive results would potentially lead to unwarranted anxiety in the parents and family, inappropriate intervention strategies and unnecessary investigations (e.g., invasive investigation involving blood drawing or brain imaging). False negatives on the other hand would lead to the failure to identify a condition and prescribe appropriate further investigations as well as interventions. Inaccurate conclusions in ascertaining risk factors could also impact clinical practice by not providing accurate risks and benefits of the specific factors associated with the condition. Apart from that, a factor that is inaccurately found not to be a risk factor and not avoided could be a catalyst leading to increased risk of ASD. Thus, accurate conclusions from well-designs and replicable studies are imperative to support optimal standards of clinical practice.

## 5. Conclusions and Future Direction

The present evidence pointed immune dysregulation, microglial activation, genetic abnormalities, mitochondrial dysfunction and mTOR signaling dysregulation as the pathogenesis of ASD. These major etiological components highlighted in this review could be a prime target for treating and preventing ASD. By understanding the biological pathways connecting genotype to phenotype, therapeutics could be applied to repair or improve affected biological pathways in ASD such as manipulation of enzymes or neurotransmitter concentrations and modulation of signaling pathway. In addition, with greater understanding of the diverse biological underpinnings of ASD, in the future, we might be able to tailor therapy for specific patients rather than using one size fits all approach. This individualized approach to medicine including for ASD could target therapy specifically to the patients, thus increasing the efficacy and reducing adverse effects. Nonetheless, further research is needed to better understand the molecular causes and how they may contribute to the pathophysiology of ASD. This information could accommodate further translation research into a treatment target for this disorder.

## Figures and Tables

**Table 1:** Summary of previous studies on brain structure and function abnormalities in autism spectrum disorder (ASD).

Reference	Subjects	Sex	Age Group	Test Samples/Regions	Method	Major Findings
[27]	7 autistic adults, 8 normal control	Male	21 to 41 years	Brain: Fusiform gyrus, inferior temporal gyrus, middle temporal gyrus, amygdala	fMRI	⇓ bilateral amygdala volumes in autistic subjects; fusiform gyrus volume was ⇓ but not statistically significant.
[28]	14 autistic subjects, 14 normal control	Male	11 to 37 years	Brain: Hippocampus, amygdala	MRI	⇓ amygdala volume (with and without total brain volume correction); ⇓hippocampal volume (with correction) in autistic subjects.
[29]	2 adults with bilateral damage to amygdala	Female	23-and 48-years	Autism Diagnostic Questionnaire Observation Schedule, Social Responsiveness Scale and other questionnaires		No evidence of autistic changes in all measurements.
[23]	45 children with ASD, 26 typically- developing (TD), 14 developmentally- delayed (DD) children	Male, female	36 to 58 months	Brain: Cerebellum, cerebrum, amygdala, hippocampus	MRI	⇑ cerebral volume in ASD compared to TD and DD children; ⇑ cerebellar volume in ASD compared to TD; ⇑ bilateral amygdala and hippocampi volume in ASD.
[30]	19 low-functioning autism (LFA), 27 high-functioning autism (HFA), 25 Asperger’s and 27 typically developing control children	Male	7.5 to 18.5 years	Brain: Amygdala, hippocampus	MRI	⇑ right and left amygdala in children with autism than control (7–12.5 years old); ⇔ amygdala volume in adolescent group (12.75–18.5 years old).
[25]	23 adolescents with autism, 29 control	Male, female	12 to 18 years hippocampus	Brain: Amygdala,	MRI	⇑ right amygdala and left hippocampus in adolescent with autism.
[31]	60 infants with risk of ASD, 211 normal control Brain: Amygdala,	Male, female	23 to 27 months	Brain: Amygdala, hippocampus	MRI	⇑ amygdala and hippocampus in each hemisphere and the whole brain in ASD group.

All main findings were compared to control; Abbreviations: ASD, autism spectrum disorder; TD: typically-developing; DD, developmentally-delayed; LFA, low-functioning autism; HLA, high-functioning autism; MRI, magnetic resonance imaging. Symbols: ⇓, decreased; ⇑, elevated; ⇔, no changes.

**Table 2:** Summary of previous studies on neuroglial activation and neuroinflammation in ASD.

Reference	Subjects	Sex	Age Group	Test Samples/Regions	Method	Major Findings
[36]	12 children with autism (group 1), 35 children with autism (group 2), 12 control	Male, female	2.7 to 10 years	CSF (group 1), serum (group 2) ELISA	ELISA	Changes in indicator of immune response (CSF: ⇑ biopterin, ⇓ quinolinic acid and neopterin, serum: ⇑ TNF receptor II in serum) in autistic children.
[37]	20 children with ASD, 20 matched control	Male	3 to 11 years	Peripheral blood mononuclear ELISA cells	ELISA	⇑ IL-13/IL10 and IFN-γ/IL-10 in children with ASD.
[38]	8 autistic patients, 8 matched control	Male, female	4 to 37 years	Frontal cortex brain tissue	Multiplex Bead Immunoassay	⇑ proinflammatory cytokines (TNF-α, IL-6 and GM-CSF), Th1 cytokines (IFN-γ) and chemokine (IL-8) in autistic patients.
[12]	11 autistic patients, 6 control	Male, female	4 to 45 years	Middle frontal gyrus, anterior cingulate gyrus, cerebellar hemisphere	Immunohisto- chemistry, protein tissue array, ELISA	Marked activation of microglia and astroglia (immunohistochemical studies); MCP-1 and TGF-β1 were the most prevalent cytokines in brain tissue (cytokine profile) of autistic patients.

All main findings were compared to control; Abbreviations: ASD, autism spectrum disorder; CSF, cerebrospinal fluid; ELISA, enzyme-linked immunosorbent assay; TNF, tumor necrosis factor; IL, interleukin; GM-CSF, granulocyte-macrophage colony-stimulating factor; IFN, interferon; MCP-1, macrophage chemoattractant protein 1; TGF-β1, transforming growth factor beta 1. Symbols: ⇓, decreased; ⇑, elevated.

**Table 3:** Summary for previous evidence on dysfunction of glutamatergic neurotransmission in ASD.

Reference	Subjects	Sex	Age Group	Test Samples/Regions	Methods	Main Findings
[50]	25 adults with ASD, 21 healthy control	Not specified	Not specified	Brain: Amygdala-hippocampal, parietal region	^1^H-MRS	⇑ amygdala hippocampal Glx but ⇔ parietal Glx of ASD subjects.
[51]	13 adults with ASD, 15 parents of ASD child (pASD), 15 healthy control	Male, female	25 to 48 years	Brain: Left and right hemisphere auditory cortex	^1^H-MRS	⇑ Glx of left and right hemisphere auditory cortex in ASD subjects, ⇔ Glx in pASD subjects.
[47]	8 children with ASD, 10 healthy control	Male, female	7 to 16.5 years	Brain: pregenual anterior cingulate cortex (pACC)	^1^H-MRS	⇑ Glx of pACC in ASD subjects.
[52]	7 adolescents with ASD, 7 healthy adolescents	Male	12 to 17 years	Brain: anterior cingulate cortex	MRS at 4T	⇑ Glu of anterior cingulate cortex in ASD.
[53]	10 children with ASD, 10 healthy control	Female	6 to 14 years	Plasma Brain: bilateral anterior cingulate, left striatum, left cerebellar, left frontal lobe	HPLC ^1^H-MRS	⇑ Glu of blood and brain in ASD subjects.
[46]	23 children with HFA, 22 healthy control	Male	8 to 17 years	Plasma	HPLC	⇑ plasma Glu, ⇓ plasma glutamine in HFA subjects.
[54]	27 children with ASD, 20 healthy control	Male, female	3 to 12 years	Platelet-poor plasma	LC-MS	⇑ plasma Glu, ⇓ plasma glutamine in ASD subjects.
[55]	18 adult autistic, 19 healthy control	Male	18 to 26 years	Serum	HPLC	⇑ serum Glu, ⇔ serum glutamine in autistic subjects.
[56]	23 autistic and Asperger’s patients with their 23 siblings and 32 parents	Male, female	4 to 29 years	Plasma	HPLC	⇑ plasma Glu, ⇓ plasma glutamine in autism or Asperger’s patients and their siblings and parents.
[60]	28 adults with ASD, 14 healthy control	Male, female	27 to 34 years	Brain: basal ganglia, dorsolateral prefrontal cortex, parietal lobe	^1^H-MRS	⇓ Glx of basal ganglia, ⇔ Glx of dorsolateral prefrontal cortex, ⇔ Glx of parietal lobe in ASD. subjects
[61]	14 adults with ASD, 14 healthy control	Male, female	21 to 50 years	Brain: anterior cingulate cortex, thalamus, temporoparietal junction, areas near or along parietal sulcus	^1^H-MRS	⇓ Glx of right anterior cingulate cortex in ASD subjects.
[62]	29 ASD patients, 29 healthy control	Male, female	26 to 44 years	Brain: anterior cingulate cortex, cerebellum	^1^H-MRS	⇓ Glx of anterior cingulate cortex but ⇔ Glx of cerebellum in ASD subjects.
[63]	26 with autism, 29 healthy control	Male	6 to 17 years	Brain: cerebral gray and white matter	^1^H-MRS	⇓ Glx of cerebral gray matter but ⇔ Glx of white matter in autistic males.
[64]	12 autistic children, 16 healthy control	Male, female	7 to 17 years	Brain: frontal lobes	^1^H-MRS	⇓ Glx/creatinine ratio of frontal lobes in autistic patients.

All main findings were compared to control; Abbreviations: ASD, autism spectrum disorder; HFA, high-functional autism; pACC, pregenual anterior cingulate cortex; ^1^H-MRS, proton magnetic resonance spectroscopy; HPLC, high performance liquid chromatography; LC-MS, liquid chromatography-mass spectrometry; Glx, glutamate and glutamine; Glu, glutamate. Symbols: ⇓, decreased; ⇑, elevated; ⇔, no changes.

**Table 4:** Summary for previous evidences on mitochondrial dysfunction in ASD.

Reference	Subjects	Sex	Age Group	Test Samples	Method	Main Findings
[74]	41 children with ASD, 41 healthy control	Male	2 to 14 years	Plasma	ELISA	Abnormal pyruvate, creatine kinase and complex I ETC in children with ASD.
[75]	10 children with ASD, 10 healthy control	Not specified	3 to 5 years	Serum	Standard method	⇑ serum lactate, AST, creatinine kinase in ASD children.
[76]	50 autistic children, 50 healthy control	Male	3 to 8 years	Serum	Auto analyzer kits from IFCC	⇑ serum lactate, AST and ALT in autistic children.
[77]	30 children with autism, 30 healthy control	Male, female	3 to 12 years	Serum	ELISA	⇓ serum coenzyme Q10 (ubiquinone) in autistic children.
[78]	15 adults with ASD, 18 typically-developed control	Male	23 to 39 years	Plasma	Capillary electrophoresis time-of-flight mass spectroscopy	⇑ plasma arginine and taurine; ⇓ oxoproline and lactic acid in ASD subject plasma.
[79]	42 autistic children, 26 healthy control	Male, female	2 to 14 years	Plasma	HPLC	⇑ plasma Glu, ⇓ plasma cyctein, tyrosine, serine, carnosine, β-alanine in autistic children.
[80]	15 autistic children, 12 non-autistic control	Male, female	2 to 17 years	Plasma	LC-MS	⇑ plasma DHA-PtdEtn, DHA-PlsEtn, saturated and poly-unsaturated VLCFA-PtdEtn in autistic children.
[81]	26 autistic patients, 26 healthy control	Not specified	4 to 12 years	Plasma	Gas chromatography	Altered fatty acid profile- ⇑ plasma levels of acetic valeric, hexanoic, stearidonic saturated fatty acids; ⇓ plasma saturated, mono, polyunsaturated fatty acids in autistic patients.
[83]	10 children with autism, 10 healthy control	Male, female	2 to 5 years	Plasma, lymphocyte	Spectrophotometry, qPCR	⇓ lymphocytes NADH oxidase, complex I activity and pyruvate dehydrogenase; ⇑ plasma pyruvate levels; ⇑ mtDNA overreplication in autistic children.
[84]	10 children with autism, 10 typically-developed control	Not specified	Not specified	Granulocytes	qPCR	⇑ mtDNA copy number and deletion in autistic children.
[85]	60 adults with ASD, 60 healthy control	Male, female	7 to 45 years	Blood	PCR	mtDNA deletions in 16.6% of ASD patients.
[86]	14 autistic children, 12 healthy control	Male, female	4 to 23 years	Brain: Frontal cortex	Colorimetric, qPCR	⇑ mtDNA gene ND1, ND4 and Cyt B ratio to nuclear DNA (⇑ mtDNA copy number); ⇓ ETC complexes 1 and V in autistic children.
[87]	Children with autism, adults with autism, age-matched control	Not specified	4 to 10 years, 14 to 39 years	Brain: Cerebellum; frontal, parietal, temporal, occipital lobes	Western Blot	⇓ levels of ETC complexes III and IV in cerebellum complexes I in frontal cortex, complexes II, III and IV in temporal cortex of autistic children; ⇔ levels of ETC complexes in adult with autism.
[88]	25 ASD patients, 20 control	Male, female	3 to 65 years	Brain: temporal Western Blot lobe		⇓ activity of ETC complexes I and IV; ⇓ protein levels of complexes I, III, IV and V in temporal lobe of ASD patients.

All main findings were compared to control. Abbreviations: ASD, autism spectrum disorder; ELISA, enzyme-linked immunosorbent assay; ETC, electron transport chain; AST, aspartate aminotransferase; ALT, alanine aminotransferase; IFCC, The International Federation of Clinical Chemistry and Laboratory Medicine; HPLC, high-performance liquid chromatography; LC-MS, liquid chromatography-mass spectrometry; Glu, glutamate; qPCR, real-time polymerase chain reaction; mtDNA, mitochondrial DNA; NADH, nicotamide adenine dinucleotide; Cyt B, cytochrome B; DHA-PtdEtn, docosahexaenoic acid–containing phosphatidylethanolamines; DHA-PlsEtn, docosahexaenoic acid – containing ethanolamine plasmalogens; VLCFA-PtdEtn, very long chain fatty acid–containing phosphatidylethanolamines. Symbols: ⇓, decreased; ⇑, elevated; ⇔, no changes.

**Table 5:** Summary for previous evidence on dysregulation of mTOR signaling pathway in ASD.

Reference	Subjects	Sex	Age Group	Test Samples	Method	Main Findings
[92]	8 children with ASD and 7 controls, 5 adolescents with ASD and 9 control	Male and female children, male adolescent	2–9 years (children) 13–19 years (Adolescent)	Postmortem-temporal lobe	Western blot	⇑ dendritic spine density, ⇓ spine pruning development in layer V pyramidal neurons; ⇑ p-mTOR level in ASD patients.
[95]	11 subjects with idiopathic autism, 13 control	Male, female	5–56 years	Postmortem-fusiform gyrus	Western blot	⇓ phosphorylated and total mTOR; ⇓ Akt, full-length TrkB, P13K, elF4B and PSD-95 in fusiform gyrus of autistic subjects.
[96]	41 children with ASD, 31 typically-developing control	Male	4–9 years	Peripheral blood mononuclear	Multiplex bead immunoassay	⇑ activity of mTOR, ERK and p70S6 kinase in T cells of ASD children.
[99]	626 individuals with ASD, 447 comparison subjects	Male, female	Not specified	Whole blood, lymphocytes	Raw microarray and clinical meta- data mega analysis	⇓ P13k-Akt-mTOR signaling cascades (diminished actin organization; ⇑ protein translation).
[100]	12 female sibling pairs, 24 male sibling pairs	Male, female	8 to 14 years	Transformed lymphoblastoid cells	RNA sequencing	⇑ mTOR-related gene set in males with ASD.

All main findings were compared to control; Abbreviations: ASD, autism spectrum disorder; mTOR, mechanistic target of rapamysin; p-mTOR, phosphorylated-mTOR; TrkB, tropomysin receptor kinase B; P13K, phosphatidylinositol 3-kinase; eIF4B, eukaryotic translation initiation factor 4B; PSD-95, postsynaptic density protein 95; ERK, extracellular receptor kinase. Symbols: ⇓, decreased; ⇑, elevated.
